# Influence of caries activity and number of saliva donors: mineral and microbiological responses in a microcosm biofilm model

**DOI:** 10.1590/1678-7757-2020-0778

**Published:** 2021-09-03

**Authors:** Chayane Souza VIANA, Tamires Timm MASKE, Cácia SIGNORI, Françoise Hélène VAN DE SANDE, Elenara Ferreira de OLIVEIRA, Maximiliano Sérgio CENCI

**Affiliations:** 1 Universidade Federal de Pelotas Programa de Pós-Graduação em Odontologia PelotasRS Brasil Universidade Federal de Pelotas, Programa de Pós-Graduação em Odontologia, Pelotas, RS, Brasil.; 2 Universidade Federal do Rio Grande do Sul Departamento de Odontologia Preventiva e Social Porto AlegreRS Brasil Universidade Federal do Rio Grande do Sul, Departamento de Odontologia Preventiva e Social, Porto Alegre, RS, Brasil.

**Keywords:** Biofilm, Microcosm, Dental caries, Demineralization, Saliva, Caries activity

## Abstract

**Objective:**

this study evaluated the mineral and microbiological response of biofilms originating from different types of saliva inoculum with distinct levels of caries activity.

**Methodology:**

the biofilms grown over enamel specimens originated from saliva collected from a single donor or five donors with two distinct levels of caries activity (caries-active and caries-free) or from pooling saliva from ten donors (five caries-active and five caries-free). The percentage surface hardness change (%SHC) and microbiological counts served as outcome variables.

**Results:**

the caries activity of donors did not affect the %SHC values. Inoculum from five donors compared to a single donor showed higher %SHC values (p=0.019). Higher lactobacilli counts were observed when saliva from caries-active donors was used as the inoculum (p=0.017). Pooled saliva from both caries activity levels showed higher mutans streptococci counts (p<0.017).

**Conclusion:**

Overall, pooled saliva increased the mineral response of the derived biofilms, but all the inoculum conditions formed cariogenic biofilms and caries lesions independently of caries activity.

## Introduction

Dental caries is a sugar-dependent disease of polymicrobial origin and has been described as one of the most prevalent human diseases.^1 - 3^ Carious lesions are formed as a consequence of complex interactions over time between an undisturbed microbial biofilm growth producing acid on the tooth surface due to a sucrose-rich diet.^4 - 6^ The continuum acid production, creating a low pH environment, is able to drive the selection of cariogenic bacteria (any microbial species capable to survival in this acidic environment) leading to a disease state and the development of caries lesion.^4 , 7^

The complexity of dental caries and the ethical issues related to its investigation in humans have led to the development of laboratory models to simulate the clinical condition under well-controlled circumstances. Several *in vitro* biofilm models have been employed to produce caries-like lesions.^8 , 9^ Despite the high variability, the microcosm biofilms originating from saliva or dental plaque inoculum have a similar capability of producing caries-like lesions and reproducing more closely the complexity of microbial changes related to cariogenic biofilm development.^10 , 11^ Saliva remains the most used inoculum source for microcosm biofilm formation due to its easy collection and handling. A recent systematic review on studies using biofilm models to develop dental caries showed that the number of donors and the caries activity profile from the donors (caries-active or caries-free) vary considerably among the studies when saliva is used as the inoculum source.^8^

The use of inoculum by mixing saliva samples from different donors may lack inherent stability, leading to unrepresentative outcomes.^12^ Moreover, when saliva inoculum from single donors is used, the biofilm variability decreases and inter-individual differences in caries-like lesions and biofilm development could be observed^13^ When sucrose regimen is applied consistently on these microcosm models, the artificial caries lesions produced tends to develop in a similar pattern for all the caries activity profiles evaluated.^10 , 14 , 15^ Azevedo, et al.^14^ (2011) and Azevedo, et al.^15^ (2014) using single-donor inoculum, provided by individuals with varying microbial profiles and caries experience, showed similar microbiological and mineral loss responses in this biofilm model, despite the caries profile of the individual donors. Similarly, Signori, et al.^10^ (2016), evaluating the cariogenic potential of biofilms originating from different sources (saliva and dental plaque) and type of inoculum (from caries-active and caries-free individuals) also showed that the cariogenic potential of biofilms was similar regardless of baseline differences between the source and type of inoculum. Although these few studies evaluated the cariogenicity of microcosm biofilms formed from different caries activity profiles, both using single salivary donors, a lack of methodological investigation into the influence of the number of inoculum (saliva) donors and the caries profile on the development of caries-like lesions was observed.

Therefore, this study aimed to evaluate the mineral and microbiological response of microcosm biofilms originating from different types of saliva inoculum (single or multiple donors) with distinct levels of caries activity (caries-active and caries-free). Two hypotheses are raised in this study: i) the caries profile of the salivary donors do not affect the potential of cariogenic biofilm formation and caries lesion development; and ii) biofilms originating from single-donor saliva have a higher cariogenic potential compared to pooled saliva from several donors.

## Methodology

Ethical approval was granted by the local Ethical Research Committee under protocol number 556.676 (Faculty of Medicine, Federal University of Pelotas – Pelotas, RS, Brazil). All volunteers included in this study were informed about the purpose of the study and signed a written informed consent. Consent was also obtained from parents or legal guardians for volunteers under 18 years old.

### Experimental design

**Figure 1:**
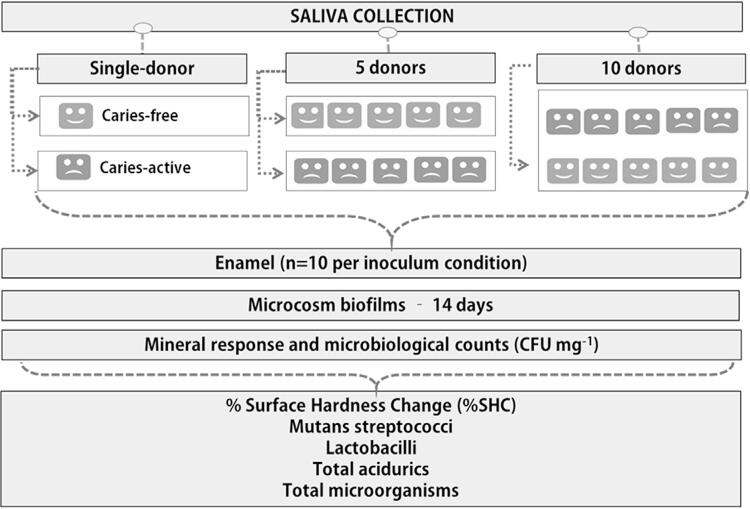
Study experimental design. Biofilms were formed over enamel specimens according to different inoculum conditions groups: single-donor (caries-free or caries-active), five donors (caries-free or caries-active) and 10 donors (five caries-free and five caries-active together)

Figure 1 shows an overview of the experimental design. Microcosm biofilms were formed individually on bovine enamel specimens (n=10 per condition) using saliva from a single donor or pooled saliva from five donors with two distinct caries activity profiles: caries-active and caries-free. Moreover, another condition consisted of pooling saliva from ten donors (five caries-active and five caries-free). In 24-well plates, the biofilms were grown over the specimens using Defined Medium with Mucin (DMM) and subjected to cariogenic challenges (DMM plus 1% sucrose) for 6 h daily up to 14 days. The primary and secondary outcome variables assessed were mineral loss (percentage surface hardness change: %SHC) and microbial composition of the biofilms (colony forming units [CFU] mg^-1^ wet biofilm): mutans streptococci, total acidurics, lactobacilli and total microorganisms.

### Sample size

The sample size was estimated based on the data of %SHC for caries-like lesion (primary outcome) published by Signori, et al.^10^ (2016). Power was set at 80% and type I error at 5%, resulting in a sample size of ten enamel specimens per saliva inoculum group (single donor, caries-free or caries-active; five donors, caries-free or caries-active; 10 donors, five caries-free and five caries-active pooled together). PS (power and sample size) software, Version 3.1.2, was used for the sample size calculation.

### Enamel specimen preparation

A minimum of 50 freshly extracted sound bovine incisors were prepared and used in this microcosm biofilm model.^16^ Enamel–dentin discs were cut from the buccal surface from each incisor using a water-cooled trephine drill. Both the dentin and the enamel surfaces were ground with 600, 1200 and 2500 grit SiC abrasive papers, respectively, to obtain plan-parallel surfaces. All procedures during disc preparation were performed under distilled water-cooling. The side and bottom surfaces of the discs were coated with nail varnish, leaving only the buccal surfaces exposed.

The baseline enamel surface hardness (SH1) was assessed by three indentations placed at the center of the enamel surface and spaced 100 μm apart using a Knoop diamond indenter loaded with a 50 g weight for 5 s (Micro Hardness Tester, FM 700, Future-Tech Corp., Tokyo, Japan). The baseline hardness of the selected enamel discs was 261.02±33.87 kgf mm^2^ (Knoop hardness number). The maximum of % variability accepted for baseline enamel surface hardness to include the enamel specimens in the study was set at 22%.

### Enamel specimen sterilization

All specimens were sterilized by gamma radiation in the Regional Center of Oncology/Radiotherapy Service (Faculty of Medicine, Pelotas, RS, Brazil). The specimens were kept moist in distilled water inside microtubes and placed 2 cm from the radiation source. They were sterilized with gamma radiation^17^ from a cobalt-60 source using particle energies of 1.25 MeV and subjected to 609.25 Gy/min. The total dose was 4.08 kGy.

### Saliva collection and inoculum saliva groups

As a general requirement, volunteers should have good general health and not have undergone antibiotic therapy in the last 6 months. In total, ten donors were selected, five caries-free and five caries-active. Volunteers included in the caries-active group should present at least two active caries lesions (cavitated or not), whereas caries-free volunteers should be free of any caries lesions. Fresh whole stimulated saliva by paraffin film chewing (6 ml) was collected from each volunteer that abstained from oral hygiene for 24 h and from food ingestion for 2 h prior to collection. No filter method was performed for the saliva collected. Saliva samples were kept in ice during collection and inoculation procedures.

Immediately after saliva collection, 1-ml aliquot of saliva from each of the five caries-free volunteers (aged 20–25 years; mean age=22.6 years) was mixed and vortexed for 1 min to create the “caries-free pooled saliva from five donors” group. The same process was performed with saliva collected from the five caries-active volunteers (aged 10–13 years; mean age=11.4 years) to create the “caries-active pooled saliva from five donors” group. One volunteer from the caries-free group and another from the caries-active group were randomly selected to represent the caries-free (aged 23 years) and caries-active single-donor (aged 13 years) inoculum, respectively. A 4-ml aliquot of saliva was collected from each one of these volunteers. Finally, 1 ml of saliva from all volunteers selected was used and pooled in a falcon tube to create the pooled saliva group with both cariogenic profile conditions (caries-active and caries-free).

### Saliva microbiological baseline measurements

After the collection procedure, an aliquot of each fresh whole saliva sample was dispersed by vortexing for 2 s, serially diluted (10^0^–10^7^ v/v) in sterile saline solution and cultivated in duplicate on the following culture media: blood agar enriched with 5% sheep/horse blood (total microorganism counts), brain heart infusion agar adjusted to pH 4.8 by few hydrochloric acid drops (total aciduric counts), *mitis salivarius agar* supplemented with 0.2 U ml^1^ of bacitracin ( *mutans streptococci* ) and *Rogosa agar* (lactobacilli). *Mutans streptococci* , total acidurics and lactobacilli were used as caries markers microorganisms. All agar plates were incubated under 5–10% CO_2_, <1% O_2_ for 96 h at 37°C. The CFU were counted and expressed as CFU ml^-1^ and used as the baseline microbiological data of the inoculum ( Table 1 ).

**Table 1:** Baseline microbiological counts according to caries activity and inoculum levels (CFU log ml -1 saliva)

Inoculum and caries activity level	Total microorganisms	Lactobacilli	Total acidurics	*Mutans streptococci*
Single donor	6,12	4,76	2,96	2,8
*caries free*				
Single donor	6,77	4,28	5,09	4,25
*caries active*				
Multi-donors - 5 donors	6,5	3,34	2,75	3,94
*caries free*				
Multi-donors - 5 donors	7,1	3,03	4,66	4,1
*caries active*				
Multi-donors - 10 donors	6,81	2,73	3,87	3,85
*5 caries free and 5 caries active*				

### Microcosm biofilm model

A microplate microcosm biofilm model was used in this study.^14 , 18^ Human saliva served as the inoculum and bovine enamel as the substratum. The nutrient growth medium used for the experiments was a defined medium enriched with 0.25% mucin (DMM) with adjusted pH 6.8 by few sodium hydroxide drops.^19^

A 0.4 ml volume of each saliva group was used to inoculate individually each enamel specimen placed in a 24-microwell plate which remained incubated at rest at 37°C. After 1 hour, 1.8 ml of defined medium enriched with mucin (DMM)^19^ containing 1% sucrose was added. The plates were incubated at 37°C under atmosphere of 5–10% CO_2_, and less than 1% O_2_. After 6 h, the samples were rinsed with 2 ml of sterile saline, inserted into a new plate containing DMM without sucrose, and incubated for 18 h under the same conditions. The biofilms were formed individually on the discs in each well for 14 days, during which the same daily routine of alternate exposure to DMM supplemented with sucrose (DMM+S) and without (DMM) was followed.^10 , 14 , 15^ The pH was daily recorded as a control procedure to monitor the experiment. The pH measurements were recorded from the supernatants (randomly from 3 wells of each group) immediately after the enamel discs were transferred to a new plate containing new medium. The supernatant pH of the DMM+S was 4.53±0.18, and 7.10±0.06 for the pure DMM.

### Outcome analyses

#### Microbiological analysis

For biofilm microbiological analysis, the enamel specimens were individually removed from each well plate and the biofilms were collected from the enamel surface with a sterile microbrush and placed in pre-weighed microtubes. The wet weight of each biofilm sample was determined, and 1 ml of sterile saline solution was added to each microtube. The biofilms were dispersed by vortexing for 1 min and sonicated for 30 s at 20 w. The biofilm suspension was serially diluted (10^0^–10^7^) and inoculated in duplicate onto the above-mentioned agar media. CFUs were counted by a trained operator blind to the study and the results were expressed as CFU mg^-1^ of biofilm (wet weight).

## Measurements of %SHC

After biofilm collection, the enamel specimens were cleaned with distilled water and brushed with a soft-bristle toothbrush. Moreover, the surface hardness was recorded for all enamel specimens by placing three indentations (SH2) spaced 100 μm to the left/right of the baseline indentations and under the same parameters as previously described. The %SHC was calculated as: %SHC = 100(SH2 – SH1)/SH1^20^ , in which SH1 refers to the baseline surface readings and SH2 to the post-biofilm surface readings.

## Statistical analysis

The effects of different inoculum conditions evaluated on the %SHC and microbiological counts (log10 CFU) were analyzed with a multivariate general linear model. SPSS software was used (Statistical Package for Social Sciences, Version 20.0, Chicago, IL, USA) and the statistical significance level was set at p<0.05.

## Results

**Figure 2:**
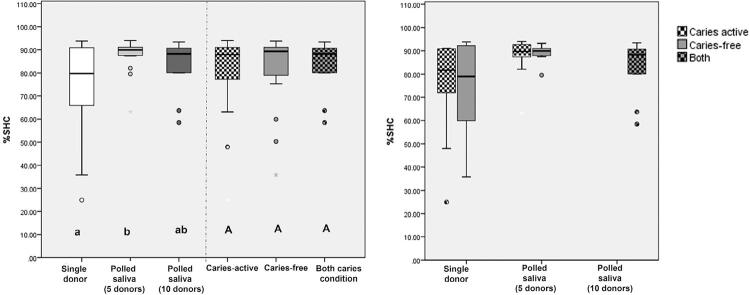
%SHC and (SD) values considering inoculum and caries activity levels (right side) and statistical set-up (left side). Upper case letters show statistical difference between caries activity level (caries free, caries active or both). Lower case letters show statistical difference among inoculum level (single donor, pooled saliva from 5 donors and 10 donors). The circles represent outliers and the asterisks extreme values

Figure 2 shows the average values for %SHC regarding each saliva inoculum group and caries activity profile condition. The %SHC values were not affected by the caries activity profile of the donors (p=0.797) but were significantly higher for the inoculum with five donors compared with a single donor (p=0.019).

Table 1 shows the baseline microbiological counts of the inoculum groups. In general, total acidurics and mutans streptococci baseline counts were lower in caries-free groups than those in carie-active donors.

**Figure 3:**
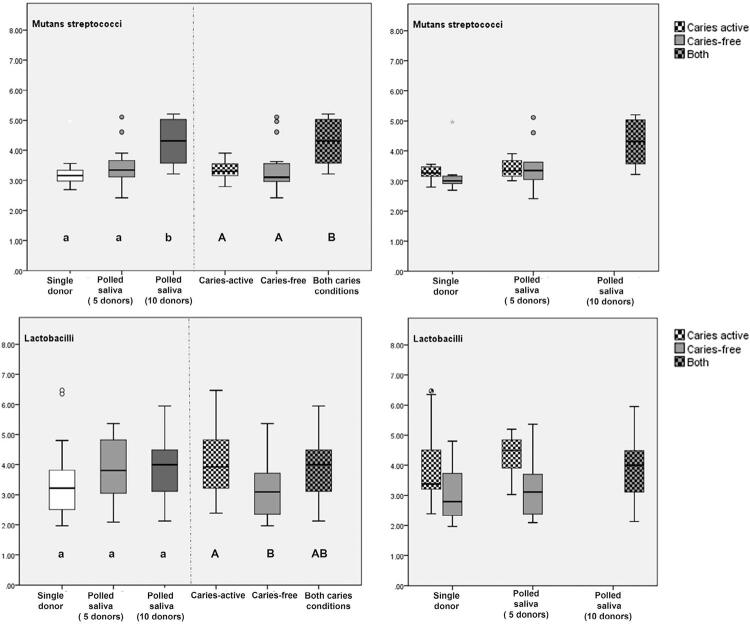
Lactobacilli and Mutans streptococci counts (CFU log mg-1) and (SD) according to caries activity and inoculum levels (right side) and statistical set-up (left side). Upper case letters show statistical difference between caries activity (caries free, caries active or both). Lower case letters show statistical difference among inoculum level (single donor, pooled saliva from 5 donors and 10 donors). The circles represent outliers and the asterisks extreme values

**Figure 4:**
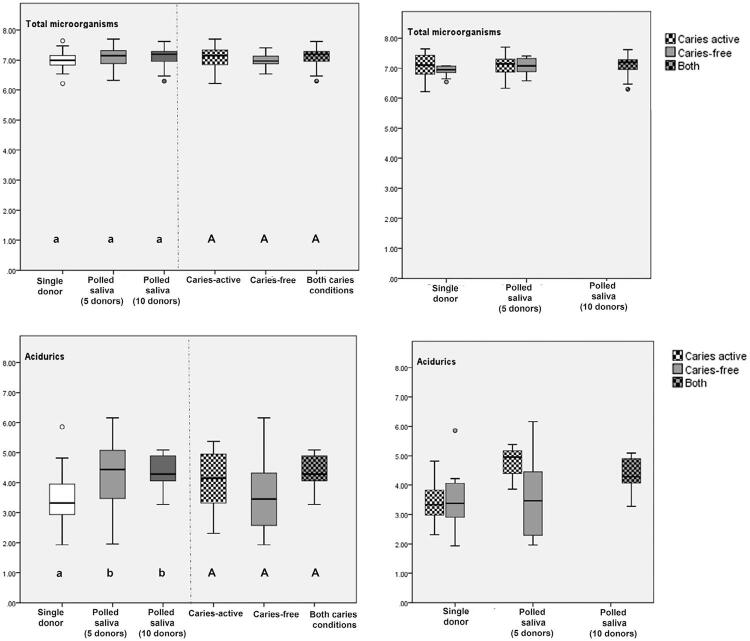
Total microorganisms and acidurics counts (CFU log mg-1) and (SD) according to caries activity and inoculum levels (right side) and statistical set-up (left side). Upper case letters show statistical difference between caries activity (caries free, caries active or both). Lower case letters show statistical difference among inoculum level (single donor, pooled saliva from 5 donors and 10 donors). The circles represent outliers and the asterisks extreme values

Figure 3 and 4 shows microbiological counts of the biofilm formed. Total acidurics counts were higher for saliva polled from five and ten donors compared to single-donor inoculum (p<0.049). Mutans streptococci counts were significantly higher for ten donors group than single and five donors (p<0.03). Similar lactobacilli counts were detected for all groups regardless the number of saliva donors.

Considering caries activity level, some significant differences in microbiological counts were found among the biofilms formed. Higher lactobacilli counts were seen when saliva from a caries-active donors were used for inoculum (caries-active and both) (p=0.017). Pooled saliva from both cariogenic profiles showed higher mutans streptococci counts compared to caries-free and caries-active groups (p=0.001). An interaction between inoculum and caries profile levels considering %SHC and microbiological data (p=0.885) was not identified.

## Discussion

This study investigated the mineral and microbiological response microcosm biofilms originating from different saliva inoculum conditions, varying in the number of saliva donors and their caries activity profile. The inoculum saliva from different caries activity profiles resulted in similar cariogenicity considering the cariogenic biofilms and caries-like lesions formation; therefore, the first hypothesis of this study was accepted. Additionally, the single-donor inoculum condition showed lower potential for caries lesion development compared to the pooled saliva condition (five or ten donors) and the second hypothesis of this study was rejected.

According to the ecological plaque theory, when biofilms are exposed to ecological pressure, such as frequent sucrose exposure, specific bacteria are selected by the acid environment.^4^ Besides, bacteria from saliva donors with or without caries activity tend to respond in a similar pattern when exposed to the same ecological pressures. Thus, regardless of the cariogenic profile, acidogenic and aciduric bacteria will be selected and dominating in the biofilm community, resulting in dental demineralization. Sucrose exposure in this model was consistently applied daily (for 14 days), which explains why caries-like lesions were developed in a similar pattern for all the caries activity profiles evaluated. These findings are in accordance with recent studies showing that there were no differences in enamel demineralization and acid-tolerant bacteria selection regardless of the caries activity of saliva donors (caries-free or caries-active).^10 , 14 , 15^

Although bovine teeth were used instead of human teeth, and no additional test were performed to confirm total biofilm detachment from enamel samples, what could generate small differences in mineral results,^21 - 23^ it was observed that pooled saliva from five donors used as the inoculum positively affected enamel demineralization when compared with single-donor inoculum. Mc Bain, et al.^12^ (2005) argued that inoculum by mixing saliva samples from different donors could lack inherent stability, leading to unrepresentative outcomes including less biofilm carcinogenicity when compared with single inoculum. On the other hand, when more donors are used to compose one inoculum, microbial interactions may occur, such as antagonism, synergism or mutualism, and this relationship may lead to differences in the demineralization pattern compared to single-donor inoculum^22^ and could explain the results of this study. In addition, demineralization response for single donor inoculum showed higher variability when compared with a different saliva inoculum (e.g., 10 saliva donors). This finding could be explained by specific bacterial pattern of the single donor used, but also as a consequence of the methodological technique used to assess the mineral response that is more appropriate to analyze superficial lesions than deeper lesion.^23^ Due to the absence of significance in some complex saliva groups, the authors suggest complementary studies considering gold-standard analysis for mineral outcomes (Transversal Microradiography [TMR] or Transversal Wavelength-independent Microradiography [T-WIM]).

In this study, the presence of higher counts of total acidurics and mutans streptocci associated with pooled saliva inoculum compared to single-donor inoculum could also be explained by microbial interactions theory. In a simulated higher cariogenic challenge, as in this study (six hour of sucrose exposition during day), acidurics and acidogenic bacteria may be more able to proliferate in response to the ecological pressure compared to a more stable microcosm represented by single donors. This is shown by comparing the baseline microbial counts of total acidurics from the caries-free group ( Table 1 ) with counts observed in the biofilms formed in the model used ( Figure 4 ). These findings show that the differences between saliva inocula may be masked by high cariogenic challenge selecting cariogenic bacteria that are more prone to survival in the acidic environment. Future studies should consider microbiome analyses to accurately analyze biofilm cariogenicity and better explain the microbiological interaction virulence factors of the biofilm (e.g., lactic acid production and extracellular polymeric substances formation).

In general, microbiological counts were not affected by the caries activity level but lactobacilli were highly expressed when using saliva from a caries-active donor. This bacterial group is often found in plaque from caries-active people and represents a marker for caries activity. Although these bacteria were not expressed in higher counts in the baseline saliva of caries-active compared to caries-free groups, they could increase considerably when exposed to a sucrose regimen.

Notwithstanding that more demineralization for the pooled saliva groups (with five or ten donors) was noticed in this model, all inocula evaluated were able to produce caries-like lesions and cariogenic biofilms in this microplate microcosm biofilm model. That is, depending on the purpose of the study, the researchers could choose the group/inoculum that best fits their study by considering the speed of results, convenience of collection, operational costs and human resources involved. Although the microcosm biofilms were formed individually over each enamel samples, it is important consider that biofilm samples corresponding to the single donor came from one volunteer (caries free or caries active profile). In this study, the statistical unit was the biofilm formed over the samples individually. Technical replicates were performed by group (n=10) to overcome possible experimental variations. The lack of biological replicates could be considered as a limitation of this study, but it was justified to decrease the human resources involved in the model and its operational costs.^24^

It is necessary to emphasize that other biofilm models, such as dynamic systems, may produce different findings if they could better represent the physiological characteristics found in the oral cavity, such as salivary flow and biofilm disturbances. Another point to be considered in this study concerns the age range of the saliva donors (10–25 years). The microbiota of individuals in different age groups may present different compositions,^25^ which is a limitation of this study. On the other hand, this biofilm model tends to standardize the responses regarding demineralization and bacteria selection.^14^

## Conclusion

All inoculum conditions could produce enamel caries-like lesions and cariogenic microcosm biofilms in the microwell model used, regardless of the caries activity profile of the saliva donors. Pooling of saliva from five or ten donors used as the inoculum increased the cariogenicity of the derived biofilms. Nevertheless, the use of a single saliva donor still remains a good option to produce caries-like lesions and cariogenic biofilms, considering the experimental factor of using only one volunteer for saliva collection and handling.
